# Hydrazines as Substrates and Inhibitors of the Archaeal Ammonia Oxidation Pathway

**DOI:** 10.1128/aem.02470-21

**Published:** 2022-04-06

**Authors:** Arne Schatteman, Chloë L. Wright, Andrew T. Crombie, J. Colin Murrell, Laura E. Lehtovirta-Morley

**Affiliations:** a School of Biological Sciences, University of East Anglia, Norwich, United Kingdom; b School of Environmental Sciences, University of East Anglia, Norwich, United Kingdom; Royal Netherlands Institute for Sea Research

**Keywords:** ammonia-oxidizing archaea, hydrazines

## Abstract

Ammonia-oxidizing archaea (AOA) and bacteria (AOB) perform key steps in the global nitrogen cycle, the oxidation of ammonia to nitrite. While the ammonia oxidation pathway is well characterized in AOB, many knowledge gaps remain about the metabolism of AOA. Hydroxylamine is an intermediate in both AOB and AOA, but homologues of hydroxylamine dehydrogenase (HAO), catalyzing bacterial hydroxylamine oxidation, are absent in AOA. Hydrazine is a substrate for bacterial HAO, while phenylhydrazine is a suicide inhibitor of HAO. Here, we examine the effect of hydrazines in AOA to gain insights into the archaeal ammonia oxidation pathway. We show that hydrazine is both a substrate and an inhibitor for AOA and that phenylhydrazine irreversibly inhibits archaeal hydroxylamine oxidation. Both hydrazine and phenylhydrazine interfered with ammonia and hydroxylamine oxidation in AOA. Furthermore, the AOA “*Candidatus* Nitrosocosmicus franklandus” C13 oxidized hydrazine into dinitrogen (N_2_), coupling this reaction to ATP production and O_2_ uptake. This study expands the known substrates of AOA and suggests that despite differences in enzymology, the ammonia oxidation pathways of AOB and AOA are functionally surprisingly similar. These results demonstrate that hydrazines are valuable tools for studying the archaeal ammonia oxidation pathway.

**IMPORTANCE** Ammonia-oxidizing archaea (AOA) are among the most numerous living organisms on Earth, and they play a pivotal role in the global biogeochemical nitrogen cycle. Despite this, little is known about the physiology and metabolism of AOA. We demonstrate in this study that hydrazines are inhibitors of AOA. Furthermore, we demonstrate that the model soil AOA “*Ca.* Nitrosocosmicus franklandus” C13 oxidizes hydrazine to dinitrogen gas, and this reaction yields ATP. This provides an important advance in our understanding of the metabolism of AOA and expands the short list of energy-yielding compounds that AOA can use. This study also provides evidence that hydrazines can be useful tools for studying the metabolism of AOA, as they have been for the bacterial ammonia oxidizers.

## INTRODUCTION

Aerobic oxidation of ammonia (NH_3_) to nitrite (NO_2_^−^) is the first step in nitrification and is carried out by ammonia-oxidizing archaea (AOA) and bacteria (AOB) along with complete ammonia-oxidizing (comammox) bacteria. Comammox bacteria and nitrite-oxidizing bacteria then further oxidize NO_2_^−^ to nitrate (NO_3_^−^). Nitrifiers play a central role in the global nitrogen cycle, with important consequences for greenhouse gas emission and leaching of nitrate from terrestrial environments. AOA are ubiquitous and significantly contribute to nitrification in many ecosystems, including acidic soils, unfertilized soils, and the oligotrophic open ocean. Ammonia monooxygenase (AMO), a member of the copper membrane monooxygenase (CuMMO) superfamily, is found in ammonia-oxidizing archaea and bacteria and initiates the nitrification process through the conversion of NH_3_ to hydroxylamine (NH_2_OH) (1, 2). Catalysis by AMO requires the input of two electrons, which are supplied by the downstream oxidation of hydroxylamine to NO_2_^−^, leaving two net electrons to enter the respiratory electron transport chain and making the oxidation of hydroxylamine the first energy-yielding reaction in the pathway (1, 3).

The functional and structural understanding of the archaeal AMO has improved through work exploring substrate analogues (4, 5). This has led to applications such as the use of octyne to distinguish between archaeal and bacterial ammonia oxidation in soil microcosms (4) and the use of alkadiynes in combination with click chemistry to label the AMO (6). AMO has never been purified in its active form, and virtually all knowledge about this enzyme comes from work on substrate analogues, highlighting the importance and potential of substrate analogues as tools in nitrification research. Very little is known about how hydroxylamine, produced from the initial oxidation of ammonia by the AMO, is converted to nitrite in archaea.

In AOB, hydroxylamine is converted to nitric oxide (NO) by hydroxylamine dehydrogenase (HAO) (7, 8), and NO is then further oxidized to NO_2_^−^ by an unknown mechanism (3). HAO is a homotrimer, with each subunit containing eight *c*-type hemes, and one of these, the active site, is a P_460_ cofactor (9). In the AOA, no genetic HAO homologue has been identified, and the genetic inventory for the production of *c*-type hemes is incomplete (10), suggesting that a fundamentally different enzyme system for hydroxylamine oxidation is required. Based on proteomics and comparative genomics, several candidate enzymes have been identified (11), including F_420_-dependent enzymes and multicopper oxidases. However, no candidate enzymes have been experimentally verified.

Hydrazine (N_2_H_4_) is an alternative substrate for HAO from AOB. It competes with hydroxylamine for access to the HAO active site and thus can be described as a competitive inhibitor (12). However, hydrazine can be used as an external source of reductants to supply the bacterial AMO with the electrons required for activity, making it possible to study the oxidation of a wide range of compounds by the AMO (13–19). The product of hydrazine oxidation by the HAO is dinitrogen gas (N_2_) (20). Anaerobic ammonia-oxidizing (anammox) bacteria also have an HAO homologue, hydrazine dehydrogenase, which catalyzes the conversion of hydrazine to N_2_ gas as part of their ammonia oxidation pathway (21).

Organohydrazines, on the other hand, are irreversible suicide inhibitors of the HAO, covalently modifying the P_460_ active site (22). Phenylhydrazine has been used to characterize the HAOs of different groups of AOB, differential responses between AOB groups (23). An attempt was made to use organohydrazines (phenylhydrazine, methylhydrazine, and 2-hydroxyethylhydrazine) to distinguish between bacterial and archaeal ammonia oxidation in soil microcosms (24), relying on the absence of a genetic HAO homologue in the AOA. However, the authors of that study found that the abundances of both AOA and AOB were affected. The inhibition of both AOA and AOB by organohydrazines was confirmed later in a different soil microcosm study (25). However, the effect of organohydrazines on AOA cultures has not been studied, and the inhibition of AOA by hydrazines warrants further investigation. If hydrazines inhibit AOA, they would be valuable tools for investigating the archaeal ammonia oxidation pathway.

The objectives of this study were to investigate the effect of hydrazines on ammonia and hydroxylamine oxidation using three strains of soil AOA and to compare the hydrazine metabolism of AOA and AOB. Specifically, we aimed to address the following questions. (i) Do hydrazines inhibit archaeal hydroxylamine and ammonia oxidation? (ii) Are hydrazines reversible or irreversible inhibitors in AOA? (iii) Are hydrazines oxidized, and do they yield ATP in AOA? (iv) Can AOA oxidize hydrazine to N_2_ as AOB do?

## RESULTS

### Effect of phenylhydrazine on NH_3_- and hydroxylamine-dependent nitrite production by ammonia oxidizers.

**FIG 1:**
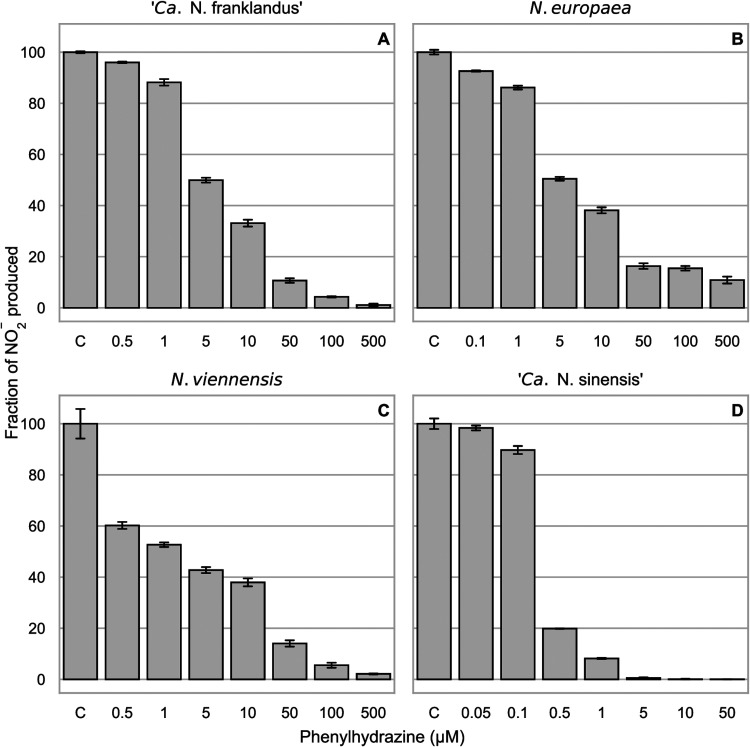
Percentage of NO_2_^−^ production compared to the uninhibited control (C) after 1 h of incubation with different concentrations of phenylhydrazine using 100 μM NH_4_^+^ as the substrate. NO_2_^−^ was measured 1 h after the addition of the substrate. Nitrite accumulation in the uninhibited control treatment represents 100% activity, and the treatments with phenylhydrazine are shown as percentages of activity compared to this control. Error bars represent standard deviations (*n* = 3). One hundred percent activity corresponded to 100 μM, 58 μM, 53 μM, and 61 μM nitrite accumulated after 1 h in (A) “*Ca.* Nitrosocosmicus franklandus,” (B) *N. europaea*, (C) *N. viennensis*, and (D) “*Ca.* Nitrosotalea sinensis,” respectively.

To investigate the effect of phenylhydrazine on NH_3_ and hydroxylamine oxidation by three different AOA strains and Nitrosomonas europaea, levels of NO_2_^−^ production, as a proxy for activity, were compared after exposure to different concentrations of phenylhydrazine (Fig. 1). Both NH_3_-dependent (Fig. 1) and hydroxylamine-dependent (Fig. S1) NO_2_^−^ production were used to characterize and compare the inhibitory thresholds of the AOA and N. europaea.

The inhibition thresholds of “*Candidatus* Nitrosocosmicus franklandus” (Fig. 1A) and *N. europaea* (Fig. 1B) were similar, but “*Ca*. Nitrosocosmicus franklandus” was less sensitive to phenylhydrazine inhibition than Nitrososphaera viennensis (Fig. 1A and C). The acidophilic AOA “*Ca*. Nitrosotalea sinensis” was more sensitive than the other AOA tested, and 5 μM phenylhydrazine inhibited NH_3_-dependent NO_2_^−^ production completely (Fig. 1D).

The inhibitory ranges of phenylhydrazine were similar between NH_3_-dependent and hydroxylamine-dependent NO_2_^−^ accumulation (Fig. 1; Fig. S1 in the supplemental material), although the threshold for phenylhydrazine inhibition was higher when hydroxylamine was used as the substrate in *N. europaea* and N. viennensis. From these results, 100 μM phenylhydrazine was chosen to inhibit all strains in subsequent experiments, except for “*Ca*. Nitrosotalea sinensis,” where 10 μM phenylhydrazine was used. The lowest concentrations that resulted in nearly full inhibition were chosen to minimize abiotic interactions and toxic effects (26). In addition, hydroxylamine is reactive and potentially toxic and may participate in abiotic reactions. To mitigate toxic effects and abiotic reactions, a suitable hydroxylamine concentration (200 μM for “*Ca*. Nitrosocosmicus franklandus” and *N. viennensis* and 100 μM for *N. europaea* and “*Ca*. Nitrosotalea sinensis”) was chosen by prescreening a range of hydroxylamine concentrations.

### Effect of hydrazine on NH_3_- and hydroxylamine-dependent NO_2_^−^ production.

**FIG 2:**
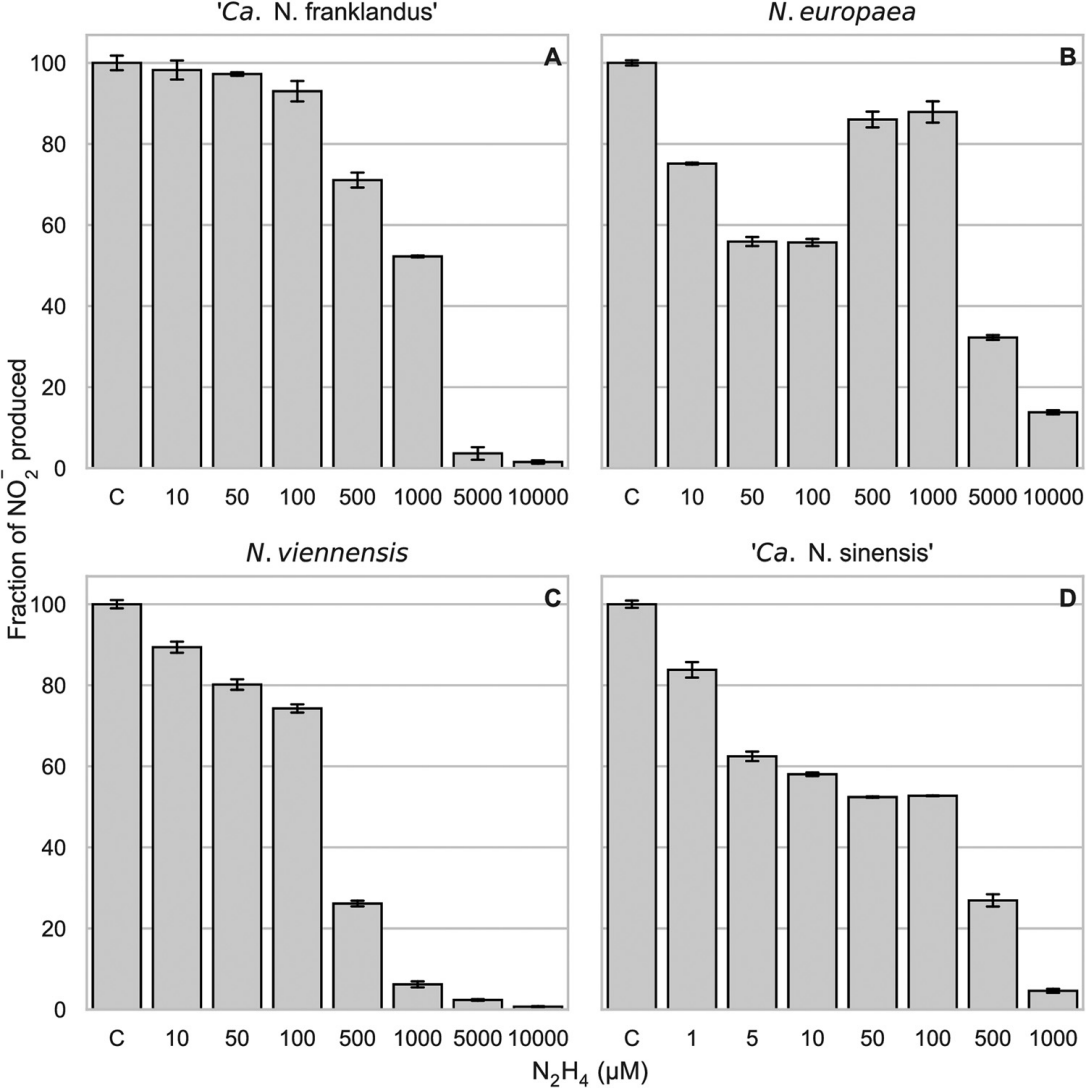
Percentage of NO_2_^−^ production compared to the uninhibited control (C) after 1 h of incubation with different concentrations of hydrazine using 100 μM NH_4_^+^ as the substrate. NO_2_^−^ was measured 1 h after the addition of the substrate. Nitrite accumulation in the uninhibited control treatment represents 100% activity, and the treatments with hydrazine are shown as percentages of activity compared to this control. Error bars represent standard deviations (*n* = 3). One hundred percent activity corresponded to 100 μM, 59 μM, 54 μM, and 63 μM nitrite accumulated after 1 h in (A) “*Ca.* Nitrosocosmicus franklandus,” (B) *N. europaea*, (C) *N. viennensis*, and (D) “*Ca.* Nitrosotalea sinensis,” respectively.

Hydrazine was tested to determine whether it inhibits NH_3_-dependent (Fig. 2) and hydroxylamine-dependent (Fig. S2) NO_2_^−^ production. It was hypothesized that hydrazine would compete with hydroxylamine as a substrate due to its similar chemical properties (27). Higher concentrations of hydrazine than phenylhydrazine were required to inhibit NO_2_^−^ production in all ammonia oxidizers (Fig. 2). In *N. europaea* hydrazine is known to be a competitive inhibitor of the HAO (12), and increasing hydrazine concentrations inhibited the ammonia oxidation activity to a greater extent (Fig. 2B). Interestingly, 500 and 1,000 μM phenylhydrazine inhibited NO_2_^−^ production less than lower concentrations in *N. europaea*. This profile was less pronounced but also apparent when hydroxylamine was used as the substrate (Fig. S2B). When supplied with NH_3_ as the substrate, the sensitivities of *N. viennensis* and “*Ca*. Nitrosotalea sinensis” to 500 μM N_2_H_4_ were very similar, but in comparison, “*Ca*. Nitrosocosmicus franklandus” was inhibited only slightly (Fig. 2A, C, and D). As with phenylhydrazine, “*Ca*. Nitrosotalea sinensis” was more sensitive to hydrazine than the other strains (Fig. 2D). The highest concentrations tested, 5,000 μM and 10,000 μM N_2_H_4_, strongly inhibited all the ammonia oxidizer strains tested and were likely toxic.

### Recovery of NO_2_^−^ production by “*Ca*. Nitrosocosmicus franklandus” following inhibition with phenylhydrazine or hydrazine.

**FIG 3:**
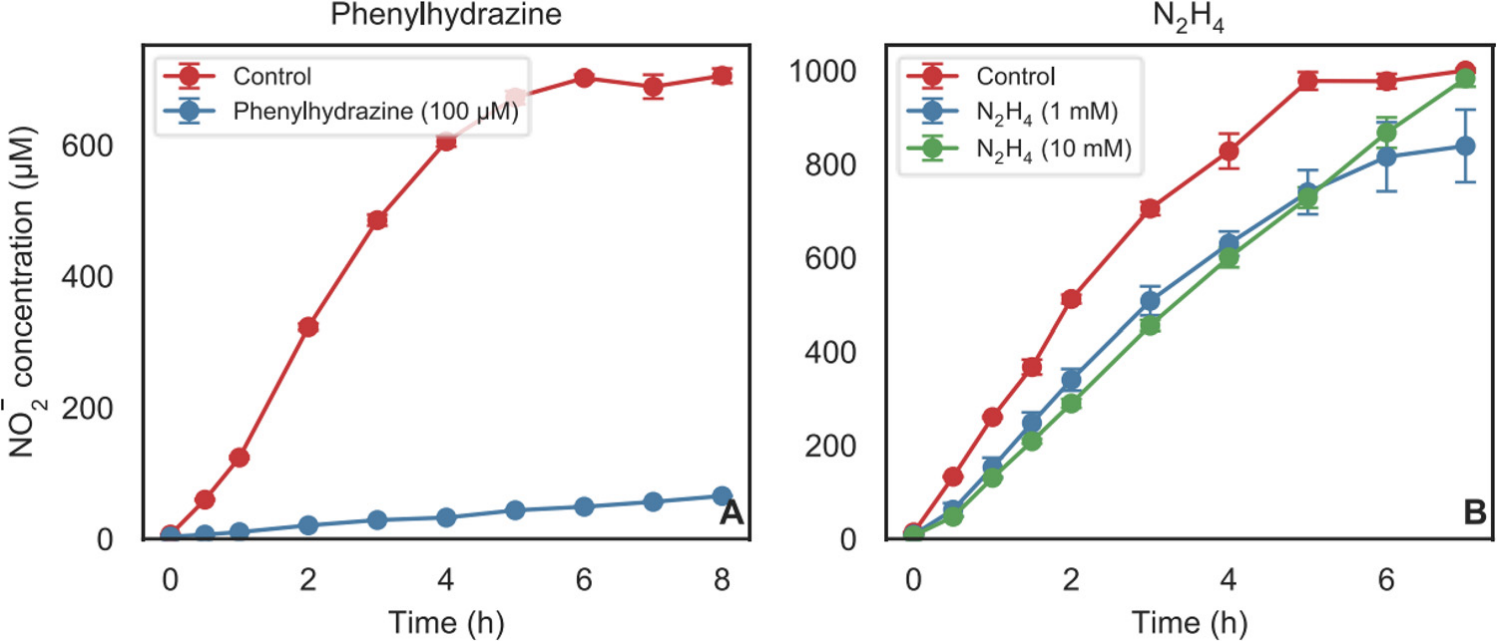
Time course of the recovery of NO_2_^−^ production from 1 mM NH_4_^+^ in “*Ca*. Nitrosocosmicus franklandus” after the removal of 100 μM phenylhydrazine (A) or 1 mM and 10 mM N_2_H_4_ (B) by washing. Error bars represent the standard deviations (*n* = 3).

Having confirmed that phenylhydrazine and hydrazine inhibited both NH_3_ oxidation and hydroxylamine oxidation in AOA and AOB, this inhibition was further characterized by testing whether phenylhydrazine and hydrazine act as reversible or irreversible inhibitors. “*Ca*. Nitrosocosmicus franklandus” was selected as the model AOA for recovery experiments with phenylhydrazine and hydrazine because of its relative ease of growth and high biomass production. Cells were treated with phenylhydrazine (100 μM) or hydrazine (1,000 μM or 10,000 μM) for 1 h, and the inhibitors were subsequently removed by washing. When an enzyme is inhibited with an irreversible inhibitor, *de novo* protein synthesis is required for the restoration of enzyme activity, resulting in a lag in recovery, as was seen after acetylene inhibition in “*Ca*. Nitrosocosmicus franklandus” (5). With a reversible inhibitor, recovery is instantaneous, as seen after 1-octyne inhibition in “*Ca*. Nitrosocosmicus franklandus” (5) and two *Nitrososphaera* species (28). “*Ca*. Nitrosocosmicus franklandus” did not recover from inhibition by 100 μM phenylhydrazine (Fig. 3A), indicating that phenylhydrazine is an irreversible inhibitor. Similarly, *N. europaea* showed no recovery when inhibited with 100 μM phenylhydrazine (Fig. S3). In contrast, inhibition with 1,000 μM and even 10,000 μM N_2_H_4_ was readily reversible in “*Ca*. Nitrosocosmicus franklandus” (Fig. 3B). This also indicates that the inhibition was not due to toxic effects but more likely due to substrate competition.

### Hydrazine-dependent O_2_ consumption in “*Ca*. Nitrosocosmicus franklandus.”

Hydrazine is an alternative substrate for the HAO in AOB and competes with hydroxylamine for the active site (12). To test if hydrazine is also a substrate for the equivalent enzyme in AOA, hydrazine-dependent O_2_ uptake by “*Ca.* Nitrosocosmicus franklandus” cells was measured and compared to hydroxylamine-dependent O_2_ uptake. Additionally, cells were preincubated with phenylhydrazine with the expectation that it would inhibit both hydrazine- and hydroxylamine-dependent O_2_ uptake. *N. europaea* was used for comparison as similar experiments have previously been performed with this nitrifier (22).

**FIG 4:**
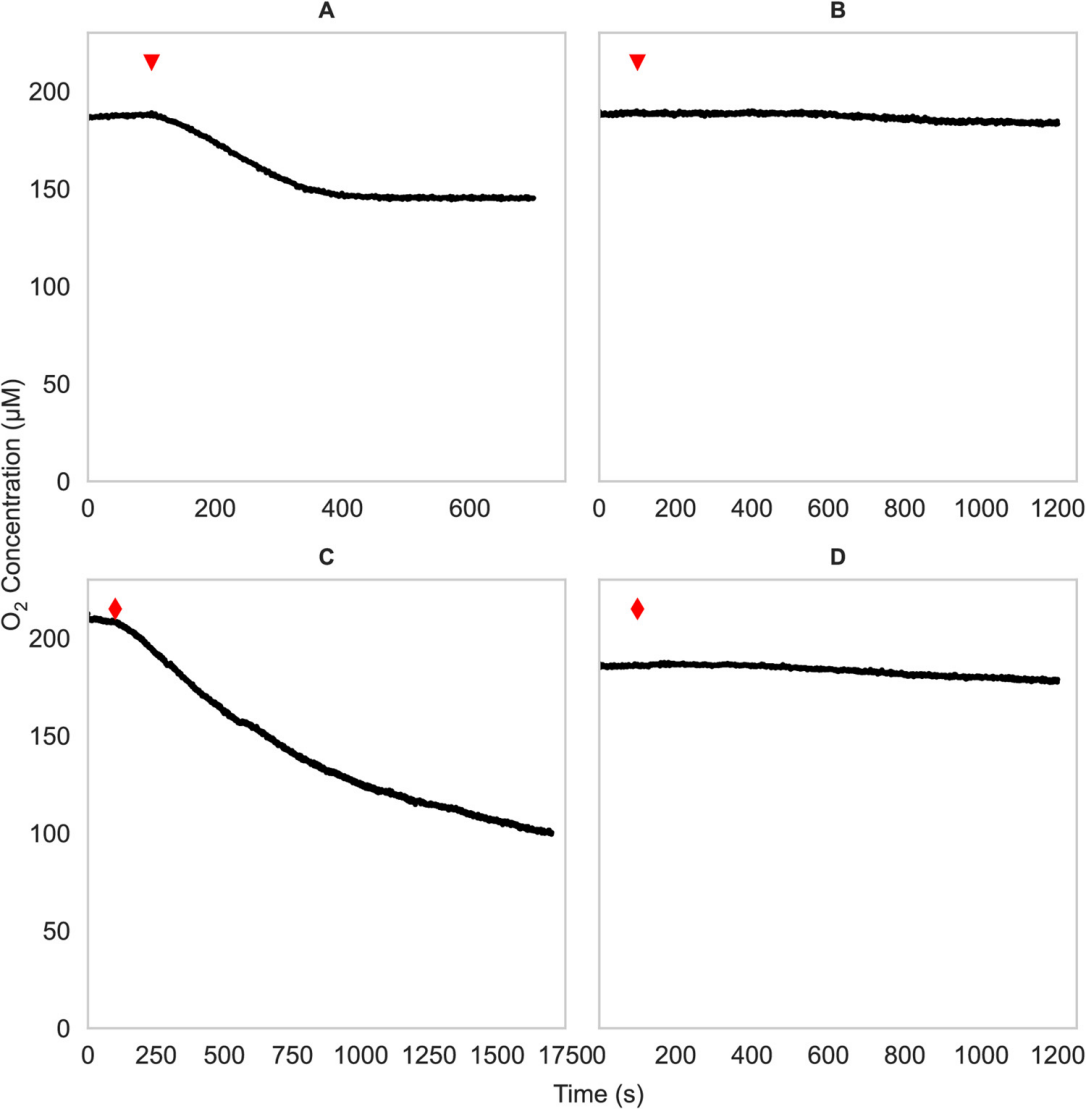
Oxygen uptake measurements in cell suspensions of “*Ca*. Nitrosocosmicus franklandus.” Concentrations of 200 μM NH_2_OH (A and B, red triangles) or 600 μM N_2_H_4_ (C and D, red diamonds) were added. Control cells (A and C) are compared to cells incubated with 100 μM phenylhydrazine (B and D). Experiments were performed at least three times with similar results.

First, hydroxylamine-induced O_2_ uptake was investigated. For “*Ca*. Nitrosocosmicus franklandus,” the optimal concentration of hydroxylamine was 200 μM since a higher concentration reduced the O_2_ uptake rate, and the induced rate was not linear (Fig. S5A). When cells were given 200 μM NH_2_OH as the substrate, “*Ca*. Nitrosocosmicus franklandus” O_2_ uptake ceased when 49 ± 4 μM O_2_ had been consumed (Fig. 4A). Subsequent spiking with 200 μM NH_2_OH caused O_2_ uptake to resume, and this could be repeated until all O_2_ was consumed (Fig. S5B), indicating that the cessation of O_2_ uptake after the reduction of 49 ± 4 μM O_2_ was due to the depletion of hydroxylamine. The consumption of 49 ± 4 μM O_2_ coincided with the production of 23 ± 1 μM NO_2_^−^, close to a 2:1 O_2_ to NO_2_^−^ stoichiometry instead of the 1:1 stoichiometry reported previously for other AOA strains (1, 29).

Second, a concentration range of 200 to 1,000 μM N_2_H_4_ was tested to determine if higher concentrations were inhibitory like hydroxylamine (Fig. S6). Only a slight increase in the initial rate was observed at concentrations over 600 μM, so for further experiments, “*Ca*. Nitrosocosmicus franklandus” cells were spiked with 600 μM N_2_H_4_, which was also chosen to allow comparison with *N. europaea*, where 600 μM N_2_H_4_ was saturating, and higher concentrations did not increase the O_2_ uptake rates (19). The initial rate of O_2_ uptake in the presence of 600 μM N_2_H_4_ in “*Ca*. Nitrosocosmicus franklandus” was 9.51 ± 0.69 μM O_2_ min^−1^, similar to the initial rate of hydroxylamine-dependent O_2_ uptake with 200 μM NH_2_OH (10.54 ± 0.21 μM O_2_ min^−1^). Notably, the hydrazine-induced rate was not linear and started to decrease after 10 min, reaching a steady rate of 1.15 μM O_2_ min^−1^ after 20 min, close to the abiotic rate (Fig. 4C). In contrast to hydroxylamine, spiking with more hydrazine did not restore the initial rate (Fig. S5C), although the addition of NH_4_^+^ caused oxygen consumption to resume (Fig. S5D). As anticipated, preincubating cells with 100 μM phenylhydrazine inhibited O_2_ consumption coupled to both hydroxylamine and hydrazine in “*Ca*. Nitrosocosmicus franklandus” (Fig. 4B and D, respectively), suggesting that the same enzyme oxidizes both substrates, as it does in *N. europaea* (20) (Fig. S4B and D), or that different enzymes are similarly affected. In contrast to AMO-specific inhibitors (e.g., acetylene (5)), phenylhydrazine inhibited hydroxylamine oxidation activity.

### ATP production in response to hydrazine and phenylhydrazine.

**FIG 5:**
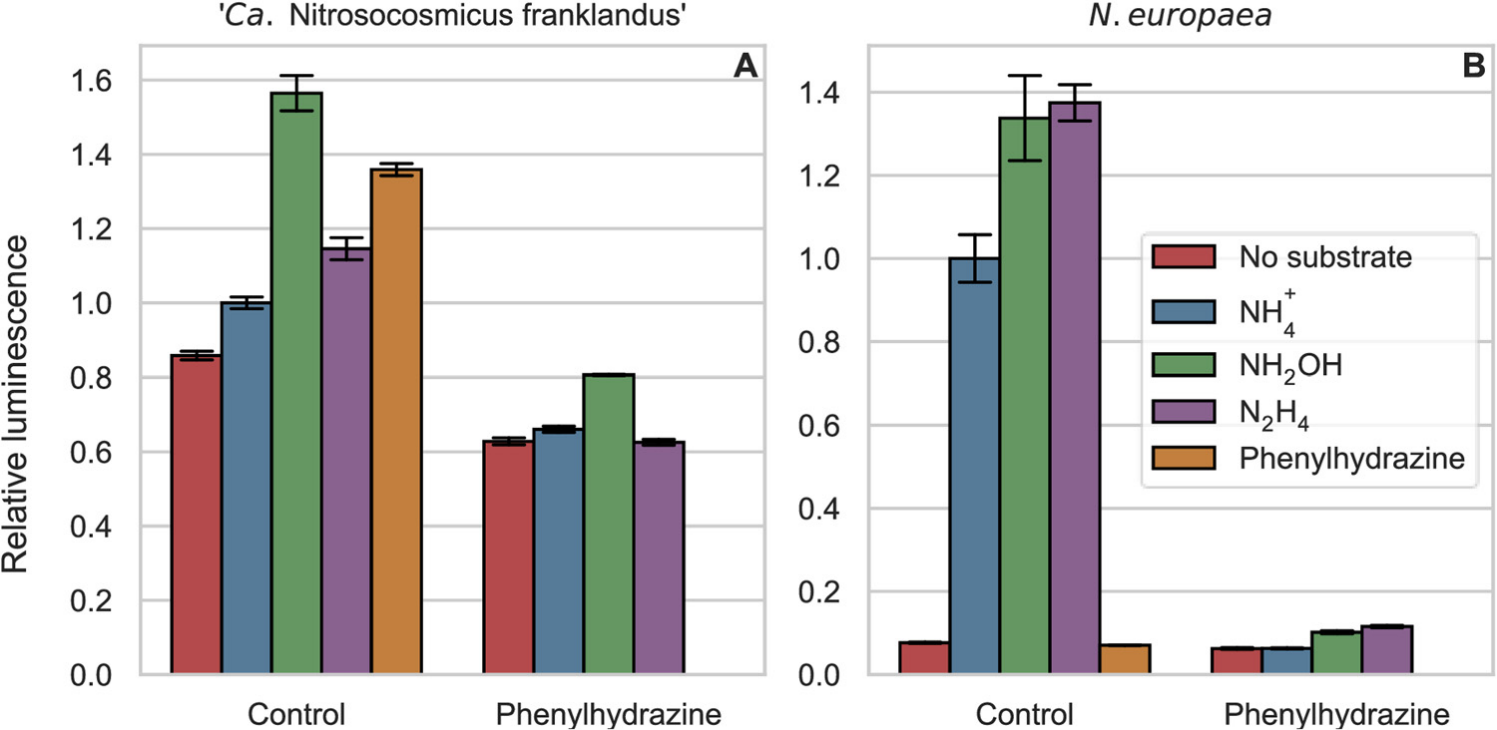
Relative ATP-dependent luminescence, with NH_4_^+^ controls normalized to a value of 1. All treatments were added in 100 μM concentrations, and the cells were incubated for 10 min before ATP was measured. Bars on the left and right of each panel show cells preincubated without and with phenylhydrazine, respectively. Error bars represent the standard deviations (*n* = 3).

To investigate whether the oxidation of hydrazines is coupled to energy conservation, ATP levels were determined in “*Ca*. Nitrosocosmicus franklandus” and *N*. *europaea*, incubated with known substrates (NH_3_ or hydroxylamine) or with hydrazine or phenylhydrazine (Fig. 5). We hypothesized that both hydroxylamine and hydrazine would yield more ATP than ammonia. The oxidation of ammonia by AMO consumes two electrons, and these electrons are normally produced from the downstream pathway of hydroxylamine oxidation. Therefore, it is expected that the net yield of electrons and ATP would be higher with hydroxylamine and hydrazine as the substrates. The higher ATP yield from hydroxylamine than from ammonia in the marine AOA Nitrosopumilus maritimus supports this notion (1). Comparisons were made between cells preincubated with and without 100 μM phenylhydrazine for 1 h. All treatments were also performed using heat-killed cells as abiotic controls, which showed no variation in ATP concentrations (data not shown).

In “*Ca*. Nitrosocosmicus franklandus,” without preincubation with phenylhydrazine, hydroxylamine yielded more ATP than NH_3_ (Fig. 5A), as was previously reported for the marine AOA N. maritimus (1). Moreover, the addition of hydrazine generated ATP in “*Ca*. Nitrosocosmicus franklandus,” confirming that hydrazine not only was oxidized but also produced energy in this AOA. The ATP concentration from hydrazine was higher than that from NH_3_ but lower than that from hydroxylamine. Unexpectedly, short-term incubations with phenylhydrazine caused an increase in ATP in “*Ca*. Nitrosocosmicus franklandus” (Fig. 5A), whereas preincubation for 1 h with phenylhydrazine depleted ATP to even lower levels than those in cells incubated with no substrate, suggesting that ATP, initially generated, was subsequently consumed and that these cells had an even greater requirement for ATP than control cells. In *N. europaea*, the amounts of ATP production in response to hydroxylamine and hydrazine were very similar (Fig. 5B). In contrast to “*Ca*. Nitrosocosmicus franklandus,” *N. europaea* produced no ATP in response to phenylhydrazine, and ATP values were comparable to those of starved cells.

### N_2_ is a product of hydrazine oxidation in “*Ca*. Nitrosocosmicus franklandus.”

**FIG 6:**
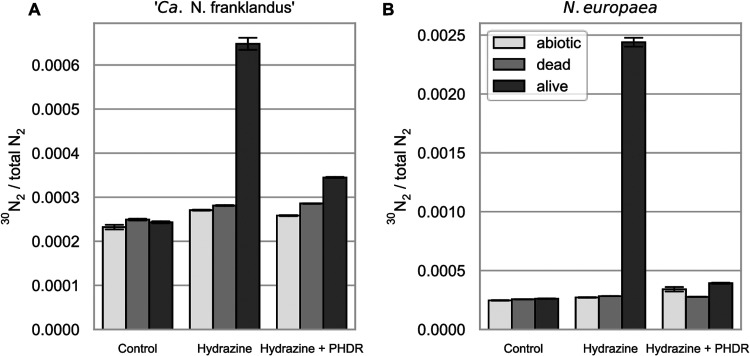
^30^N_2_/total N_2_ ratio after 1 h of incubation of cell suspensions of “*Ca*. Nitrosocosmicus franklandus” (A) and *N. europaea* (B) with 500 μM ^15^N-labeled N_2_H_4_. Abiotic and dead cells were included as controls. Error bars represent the standard deviations (*n* = 3); PHDR, phenylhydrazine.

To test if N_2_ is a product of hydrazine oxidation in the AOA as it is in the AOB (20), ^15^N-labeled hydrazine was added to cell suspensions of “*Ca*. Nitrosocosmicus franklandus” and *N. europaea*. As expected, ^29^N_2_/^28^N_2_ ratios of the abiotic, killed control, and live-cell incubations did not differ from the natural abundance in the atmosphere. In contrast, there was a clear enrichment of ^30^N_2_ with both “*Ca*. Nitrosocosmicus franklandus” C13 (Fig. 6A) and *N. europaea* (Fig. 6B), indicating that both organisms produced ^30^N_2_ when ^15^N-hydrazine was added. Additionally, the production of ^30^N_2_ from ^15^N-hydrazine was inhibited when 100 μM phenylhydrazine was included in the incubations. From the total 1,500 nmol of ^15^N-hydrazine added, 129 (±2) nmol and 1,049 (±23) nmol of ^30^N_2_ were produced by “*Ca*. Nitrosocosmicus franklandus” and *N. europaea*, respectively, during the 1-h incubation. This is equal to ∼8.8% and 71.4% of the total added ^15^N-hydrazine being oxidized to ^30^N_2_ by “*Ca*. Nitrosocosmicus franklandus” and *N. europaea*, respectively. This is consistent with the oxygen electrode data described above (Fig. 4A and C) and suggests that *N. europaea* was able to consume most of the ^15^N-hydrazine during the experiment, whereas “*Ca*. Nitrosocosmicus franklandus” was able to oxidize hydrazine only transiently. It is likely that 71.4%, rather than 100%, of the hydrazine was recovered as N_2_ in *N. europaea* due to abiotic degradation of hydrazine.

## DISCUSSION

### Hydrazines as inhibitors of AOA.

While the enzyme that catalyzes hydroxylamine oxidation in AOA has not been identified, hydrazine and phenylhydrazine seem to have similar effects on NH_3_ and hydroxylamine oxidation by AOA and AOB. Phenylhydrazine and hydrazine inhibit hydroxylamine and NH_3_ oxidation in AOA as they do in the AOB *N. europaea*. The variability in inhibition thresholds between different AOA and AOB strains clearly demonstrates the value of using more than one model microorganism, preferably from different clades, when evaluating inhibitors, as has been described previously with AOB (30). A recent study revealed a pattern in the affinity for ammonia similar to the one found for the sensitivity to hydrazines in this study (31), indicating that these different sensitivities may reflect the niche in the environment, with the organisms with higher ammonia affinities being more sensitive to inhibition by hydrazines.

The inhibition of ammonia oxidation by hydrazine has been extensively studied in AOB and, more specifically, *N. europaea* (2, 7, 32). Hydrazine is a reversible competitive inhibitor of the HAO enzyme (12), which explains the inhibition of NO_2_^−^ production by *N. europaea* at higher concentrations (Fig. 2B). However, the inhibition pattern where 500 to 1,000 μM hydrazine inhibited NO_2_^−^ production in *N. europaea* less than hydrazine concentrations below 500 μM is difficult to explain and is probably caused by an interplay of hydrazine-driven NO_2_^−^ reduction (33), the abundance of the reductant (19), and interactions of the HAO and cytochrome P_460_ with hydrazine (34).

In AOA, hydrazine interfered with NO_2_^−^ production from both NH_3_ and hydroxylamine, likely due to competition with hydroxylamine for the substrate binding site. It was not possible to unequivocally demonstrate competitive inhibition as hydroxylamine concentrations of >200 μM reduced the hydroxylamine oxidation rate (see Fig. S5A in the supplemental material). The inhibition with hydrazine in AOA was readily reversible in a manner similar to that for AOB.

Organohydrazines were characterized in *N. europaea* as irreversible suicide substrates of the HAO enzyme using purified protein (22). They have been successfully used to inhibit both archaeal and bacterial nitrification in soil microcosms (24, 25), and here, we provide insight into the archaeal inhibition mechanism. Phenylhydrazine would likely have a short-lived inhibitory effect on nitrification due to its role as an irreversible inhibitor of AOA and AOB. However, the use of phenylhydrazine as an HAO-specific nitrification inhibitor for long-term experiments (>24 h) should be cautioned as it is sensitive to light and unstable in aqueous solutions (26). Nevertheless, it is interesting to consider that hydrazine and its derivatives could potentially enter soil environments either through anthropogenic routes or, to a much lesser extent, via biological production by diazotrophs and some fungi. While the potential for environmental applications and the impacts of hydrazines are unknown, at least in theory, it may be possible that AOA could oxidize hydrazine and gain ATP from this reaction.

While it was not possible to use pure enzymes from AOA, phenylhydrazine was identified as an inhibitor of hydroxylamine oxidation in the three tested AOA strains. The inhibition specific to hydroxylamine/hydrazine oxidation was verified and characterized in “*Ca.* Nitrosocosmicus franklandus” using several approaches, including NO_2_^−^ accumulation assays, oxygen consumption, and ATP assays. As in the AOB, inhibition by phenylhydrazine in “*Ca*. Nitrosocosmicus franklandus” was irreversible, and the cells did not recover, even after several hours (5, 22). Consistent with these approaches, the production of ^15^N-labeled N_2_ was also inhibited by phenylhydrazine. One caveat is that it was impossible to verify that phenylhydrazine inhibits the hydroxylamine oxidation enzyme directly, and it could also be affecting downstream enzymes.

### Hydrazine as a substrate in AOA.

Oxygen consumption in response to hydrazine confirmed that hydrazine was used as a substrate. However, hydrazine-induced oxygen uptake decreased over time (Fig. 4C), indicating that an unknown mechanism limits the oxidation of hydrazine. The product of hydrazine oxidation is likely N_2_, as it is in AOB (20), making product inhibition unlikely. The incubations with ^15^N-labeled hydrazine confirmed that N_2_ is a product of hydrazine oxidation in the AOA, but it is possible that there are other products such as NO or N_2_O.

ATP was produced after hydrazine addition in AOA, and future studies should investigate the use of hydrazine in growth experiments and as a source of reductants in physiological experiments. In both *N. europaea* and “*Ca*. Nitrosocosmicus franklandus,” hydroxylamine and hydrazine produced higher ATP concentrations than NH_3_, which was expected. However, in *N. europaea*, the amounts of ATP produced from both substrates were similar, while in “*Ca*. Nitrosocosmicus franklandus,” they were vastly different. This could be due to differences in the NH_3_ oxidation pathways in AOA and AOB or differences in the rates of oxidation, as hydrazine had a lower initial oxidation rate in the AOA, and the rate decreased over time (Fig. 4C). Interestingly, ATP values increased in “*Ca*. Nitrosocosmicus franklandus” after short-term incubations with phenylhydrazine, which was not observed in the AOB. A possible explanation is that phenylhydrazine can serve as a substrate, but its product, or phenylhydrazine itself, becomes toxic. Hemoproteins such as the HAO of AOB are thought to be inhibited by organohydrazine derivatives by the formation of a cation radical (22). It is likely that a similar radical is formed in the AOA.

### Outlook for applications of hydrazines in ammonia oxidation research.

The fact that the hydrazine inhibitors affect AOA and AOB could aid in the development of the next generation of nitrification inhibitors. The availability of crystal structures for the bacterial HAO provides an advantage in the development of new nitrification inhibitors, compared to the AMO, for which no structures are available (9, 23). It is important, however, not to overlook other nitrifiers such as AOA and comammox bacteria in these studies but to investigate further potential inhibitors that target all ammonia oxidizers.

While it is known that organohydrazines inhibit a broad range of enzymes with both oxidative (22, 35) and electrophilic (36) cofactors via a covalent mechanism, it is interesting that they inhibit hydroxylamine oxidation in AOA, which are thought not to have the same heme cofactor as AOB. The inhibition of the archaeal hydroxylamine oxidation mechanism by organohydrazines could be used to finally identify this long-sought-after enzyme by using either ^14^C-phenylhydrazine (37) or hydrazine probes as activity-based protein profiling probes (38, 39).

In conclusion, this study provides evidence that hydrazine can be oxidized by AOA and that N_2_ is a product of its oxidation. Phenylhydrazine was shown to be an inhibitor of archaeal hydroxylamine oxidation. The inhibition by hydrazines was tested in several environmentally relevant strains of terrestrial AOA, and further characterization was done in “*Ca*. Nitrosocosmicus franklandus.” We demonstrate that (i) hydrazine and phenylhydrazine inhibit archaeal NH_3_ and hydroxylamine oxidation at concentrations comparable to those in AOB and (ii) hydrazine acts as a reversible inhibitor and phenylhydrazine acts as an irreversible inhibitor in both AOA and AOB. Furthermore, we demonstrate that (iii) hydrazine is oxidized by AOA, and this reaction yields ATP, and (iv) N_2_ is produced from hydrazine oxidation by AOA, as is the case in AOB and anammox bacteria. Despite the profound differences in the enzymology of archaeal and bacterial NH_3_ oxidation pathways, this study demonstrates that hydrazine metabolism in AOB and AOA is surprisingly similar: both AOA and AOB were inhibited by hydrazines in similar manners and with similar thresholds, and both groups of ammonia oxidizers were able to generate ATP by oxidizing hydrazine to N_2_. Future studies should focus on identifying the enzymes affected by phenylhydrazine and the use of hydrazine in growth experiments and as a source of reductants.

## MATERIALS AND METHODS

### Chemicals.

Research-grade phenylhydrazine (C_6_H_5_NHNH_2_), N_2_H_4_, and NH_2_OH (>99%, 97%, and >99%, respectively) were obtained from Sigma-Aldrich (CAS no. 59-88-1, 2644-70-4, and 5470-11-1, respectively). ^15^N-hydrazine sulfate (>98% purity) was purchased from Cambridge Isotope Laboratories (CAS no. 88491-70-7). Aqueous stock solutions of N_2_H_4_, NH_2_OH, and phenylhydrazine were prepared fresh for every experiment.

### Growth of microorganisms.

“*Candidatus* Nitrosocosmicus franklandus” C13 and Nitrososphaera viennensis EN76^T^ were cultivated in HEPES-buffered (pH 7.5) freshwater medium (FWM) (37) and supplied with 4 mM and 2 mM NH_4_Cl, respectively. In addition, 0.5 mM sodium pyruvate and 50 mg L^−1^ kanamycin were added to the *N. viennensis* FWM (40). The acidophilic AOA “*Candidatus* Nitrosotalea sinensis” Nd2 was cultivated in morpholineethanesulfonic acid (MES)-buffered FWM (pH 5.3) as previously described (41) and supplied with 400 μM NH_4_Cl. All three AOA strains were grown in 800-mL volumes in acid-washed 1-L Duran bottles and incubated statically at 37°C in the dark. Nitrosomonas europaea ATCC 19718 was obtained from the University of Aberdeen Culture Collection and cultivated in phosphate-buffered medium (pH 7.8) containing 50 mM NH_4_^+^, as described previously (42). *N. europaea* was incubated with shaking (160 rpm) in 1-L or 100-mL culture volumes in conical flasks (2 L and 250 mL, respectively) at 30°C in the dark. The purity of the cultures was monitored by microscopy and screening for contaminants on R2A agar plates (Oxoid, Basingstoke, UK). Cell counts were performed as described previously (41).

### Nitrite determination.

The growth and activity of the cultures were monitored by nitrite accumulation. The nitrite concentration was determined using a colorimetric assay with Griess reagent in a 96-well plate format (41). The detection limit was <1 μM. The absorbance was measured at 540 nm using a VersaMax microplate reader (Molecular Devices, CA, USA).

### Cell harvesting and preparation.

For “*Ca*. Nitrosocosmicus franklandus” and *N. viennensis*, 800 mL of a mid- to late-exponential-phase culture (corresponding to 1,000 to 1,500 μM NO_2_^−^ accumulation) was harvested via filtration using a 0.22-μm-pore-size polyethersulfone (PES) membrane filter (Millipore). “*Ca*. Nitrosocosmicus franklandus” cells were washed three times on the filter using 100 mL 10 mM HEPES-buffered FWM salts (pH 7.5) to remove residual nitrite and ammonia. Harvested cells were resuspended by turning over the filter and flushing it with a fresh solution to dislodge the cells. *N. viennensis* cells adhered more strongly to the filter than the other strains and were scraped from the filter after filtration and subsequently washed three times in 100 mL 10 mM HEPES-buffered FWM salts (pH 7.5) by centrifugation (10 min at 4,000 × *g*) before finally being resuspended in fresh HEPES-buffered FWM salts. For “*Ca*. Nitrosotalea sinensis,” 1.6 L of a mid- to late-exponential-phase culture (100 to 200 μM NO_2_^−^) was used. The cells were washed three times on the filter with 100 mL 2.5 mM MES-buffered FWM salts (pH 5.3) and resuspended in the same solution. For *N. europaea*, 100 mL of a mid-exponential-phase culture (15 to 18 mM NO_2_^−^) was harvested by filtration, washed three times with 100 mL 50 mM sodium phosphate buffer (pH 7.8) containing 2 mM MgCl_2_, and resuspended in the same buffer. After harvesting, the cells were incubated at their respective growth temperatures for 1 h to consume any remaining ammonia. Background NO_2_^−^ was measured prior to commencing the experiments to determine the baseline levels. All subsequent experiments were performed as short-term activity assays (<24 h in duration) rather than growth assays.

### Endpoint inhibition assays.

To evaluate the inhibition of ammonia and hydroxylamine oxidation by hydrazine and phenylhydrazine, a 96-well microtiter plate was prepared with 5 μL of the inhibitor from concentrated aqueous stocks or 5 μL of double-distilled water (ddH_2_O) (controls) in each well. The inhibitors were diluted to final concentrations of 1 to 10,000 μM hydrazine and 0.05 to 500 μM phenylhydrazine with 95 μL of a cell suspension (∼2 × 10^8^, ∼3 × 10^8^, ∼9 × 10^8^, and ∼2 × 10^8^ cells mL^−1^ for “*Ca*. Nitrosocosmicus franklandus,” *N. europaea*, *N. viennensis*, and “*Ca*. Nitrosotalea sinensis,” respectively) (see Table S1 in the supplemental material). The plate was preincubated with the inhibitors at the respective growth temperatures for 1 h, before 2 μL of the substrate (either ammonia or hydroxylamine) was added to each well and the plate was incubated for one more hour. Griess reagent was added to stop the reaction and to determine the nitrite concentration. Background nitrite, before substrate addition, was subtracted, and nitrite production was normalized to the control. Hydroxylamine-dependent NO_2_^−^ accumulation in AOA is not stoichiometric with hydroxylamine consumption (29), and the threshold for hydroxylamine toxicity in AOA is lower than that in AOB, leading to low levels of NO_2_^−^ accumulation in AOA with this substrate. Therefore, the absolute, rather than relative, values of NO_2_^−^ accumulation are shown for experiments with hydroxylamine as the substrate. The final ammonia concentration was 100 μM, and the final hydroxylamine concentrations were 100 μM for *N. europaea* and “*Ca*. Nitrosotalea sinensis” and 200 μM for “*Ca*. Nitrosocosmicus franklandus” and *N. viennensis*. Each assay was done at least two times with similar results with three biological replicates for each treatment.

### Oxygen consumption experiments.

A Clark-type electrode (Rank Brothers, Cambridge, UK) was used to determine substrate-induced oxygen consumption. The instrument was comprised of a 3-mL reaction chamber, which was sealed with a stopper containing an injection port. The temperature was maintained by a circulating water bath (Churchill Co. Ltd., Perivale, UK). The temperature was set to the growth temperature of each microorganism, and the electrode was calibrated as described previously (43). The polarizing voltage was set to 0.6 V. Cell suspensions (3 mL) were fully oxygenated by stirring for 5 min without the stopper. The chamber was then sealed, the endogenous rate was established for 2 to 5 min, and the substrate was injected in 15-μL volumes from freshly made concentrated aqueous stocks of NH_4_^+^, NH_2_OH, or N_2_H_4_ (Table S2). Endpoint inhibition assays informed the choice of the substrate and inhibitor concentrations for the O_2_ uptake experiments. Experiments were carried out with either uninhibited cells or cells preincubated with 100 μM phenylhydrazine (∼7 × 10^8^ and ∼3 × 10^8^ cells mL^−1^ for “*Ca*. Nitrosocosmicus franklandus” and *N. europaea*, respectively). Nitrite was measured at the end of each O_2_ uptake trace. Abiotic controls for all treatments were performed using buffered salts without cells, and oxygen consumption never exceeded 0.5 μM min^−1^. Reactions were performed in triplicate with similar results.

### ATP assays.

To assess the effects of hydrazine and phenylhydrazine on ATP production, cells (∼2 × 10^8^ and ∼3 × 10^8^ cells mL^−1^ for “*Ca*. Nitrosocosmicus” and *N. europaea*, respectively) were first washed and starved without any substrates for 1 h to deplete internal ATP levels. For killed controls, cells were autoclaved at 121°C for 15 min. Pretreatment of cells, where necessary, was performed by incubation for 1 h with 100 μM phenylhydrazine at the respective growth temperatures. Experiments were performed in opaque black 96-well plates, which were prepared with 5 μL of 20-times-concentrated substrate/inhibitor stocks (100 μM final concentration) and 95 μL of a live- or dead-cell suspension. The cells were mixed with the different inhibitors and substrates by pipetting and then incubated for 10 min at the respective growth temperatures. ATP accumulation was then measured using a luminescence assay based on the luciferase enzyme (BacTiter-Glo; Promega, WI, USA). BacTiter-Glo reagent (100 μL) was added, and luminescence was measured every 5 min using a Spectramax ID5 plate reader (Molecular Devices, CA, USA) with a 1-s integration time. The luminescence values reached their maxima after 10 min and remained stable for 5 to 10 min thereafter. Therefore, fluorescence was measured 10 min after the addition of the BacTiter-Glo reagent. The data were normalized against the NH_4_^+^ control (100%) (0.1 mM NH_4_Cl). Killed controls were all very similar and were subtracted from their respective live measurements. Each assay was done at least two times with similar results and with three biological replicates for each treatment.

### Recovery assay.

“*Ca*. Nitrosocosmicus franklandus” and *N. europaea* were harvested, washed, and starved as described above (∼2 × 10^8^ cells mL^−1^ and ∼2 × 10^8^ cells mL^−1^, respectively). Aliquots of 5-mL cell suspensions were added to acid-washed 23-mL glass vials, and inhibitors were added from concentrated aqueous stocks to final concentrations of 100 μM phenylhydrazine and 1,000 μM or 10,000 μM N_2_H_4_. The hydrazine concentrations were chosen to include an inhibitory but nontoxic concentration (1,000 μM) and a fully inhibitory concentration that might be toxic (10,000 μM). The vials were sealed with twice-autoclaved butyl rubber seals and incubated for 1 h at the respective growth temperatures of the organisms. After inhibition, the cells were washed by filtration and resuspended in their respective media supplied with 1 mM NH_4_Cl. The vials were then incubated for 8 h, and NO_2_^−^ was measured every 30 min for the first 2 h and every 60 min thereafter. All treatments were performed in triplicate.

### ^15^N stable isotope analysis.

To determine if N_2_ is a product of hydrazine oxidation, ^15^N-hydrazine sulfate was added to a concentration of 500 μM to cell suspensions as described above, and ^29^N_2_ and ^30^N_2_ production was investigated. If N_2_ is produced from ^15^N-labeled hydrazine, an enrichment in ^30^N_2_ is expected as ^29^N_2_ cannot be a product of this reaction. “*Ca*. Nitrosocosmicus franklandus” and *N. europaea* were harvested, washed, and rested as described above (∼3 × 10^8^ and ∼4 × 10^8^ cells mL^−1^, respectively). For abiotic controls, samples containing just the medium components as well as heat-killed cells (121°C for 15 min) were included. Additionally, phenylhydrazine-treated samples (100 μM for 1 h) were included to see if this would inhibit ^30^N_2_ production. Incubations were carried out in triplicate. ^15^N-labeled hydrazine sulfate was added from a concentrated aqueous stock, after which the vials were sealed with twice-autoclaved butyl rubber seals and incubated for 1 h at the respective growth temperatures of the organisms. The vials were shaken (180 rpm) to ensure gas exchange between the liquid and the headspace. Headspace gas (10 mL) was then sampled using a gastight syringe fitted with a Luer lock and injected into preevacuated (<0.1 atm) 12-mL exetainers (Labco). Gas samples were analyzed using a Sercon CryoPrep gas concentration system interfaced with a Sercon 20-20 isotope ratio mass spectrometer (Stable Isotope Facility, University of California, Davis, CA, USA). Molar fractions were calculated from the isotope ratios and the N_2_ concentration in the vials. The amount of ^30^N_2_ in the liquid was calculated using Henry’s law (44) and standard conditions and was added to the total ^30^N_2_ formed. The background amount of ^30^N_2_ from heat-killed cells was subtracted.
