# Supplementary material for: Hydrazines as Substrates and Inhibitors of the Archaeal Ammonia Oxidation Pathway
Source: Appl Environ Microbiol. 2022 Apr 6;88(8):e02470-21. doi: 10.1128/aem.02470-21 (PMC9040604; doi:10.1128/aem.02470-21)
Supplement: Supplemental file 1 — Fig. S1 to S6 and Tables S1 and S2. Download aem.02470-21-s0001.pdf, PDF file, 2.2 MB [file aem.02470-21-s0001.pdf]

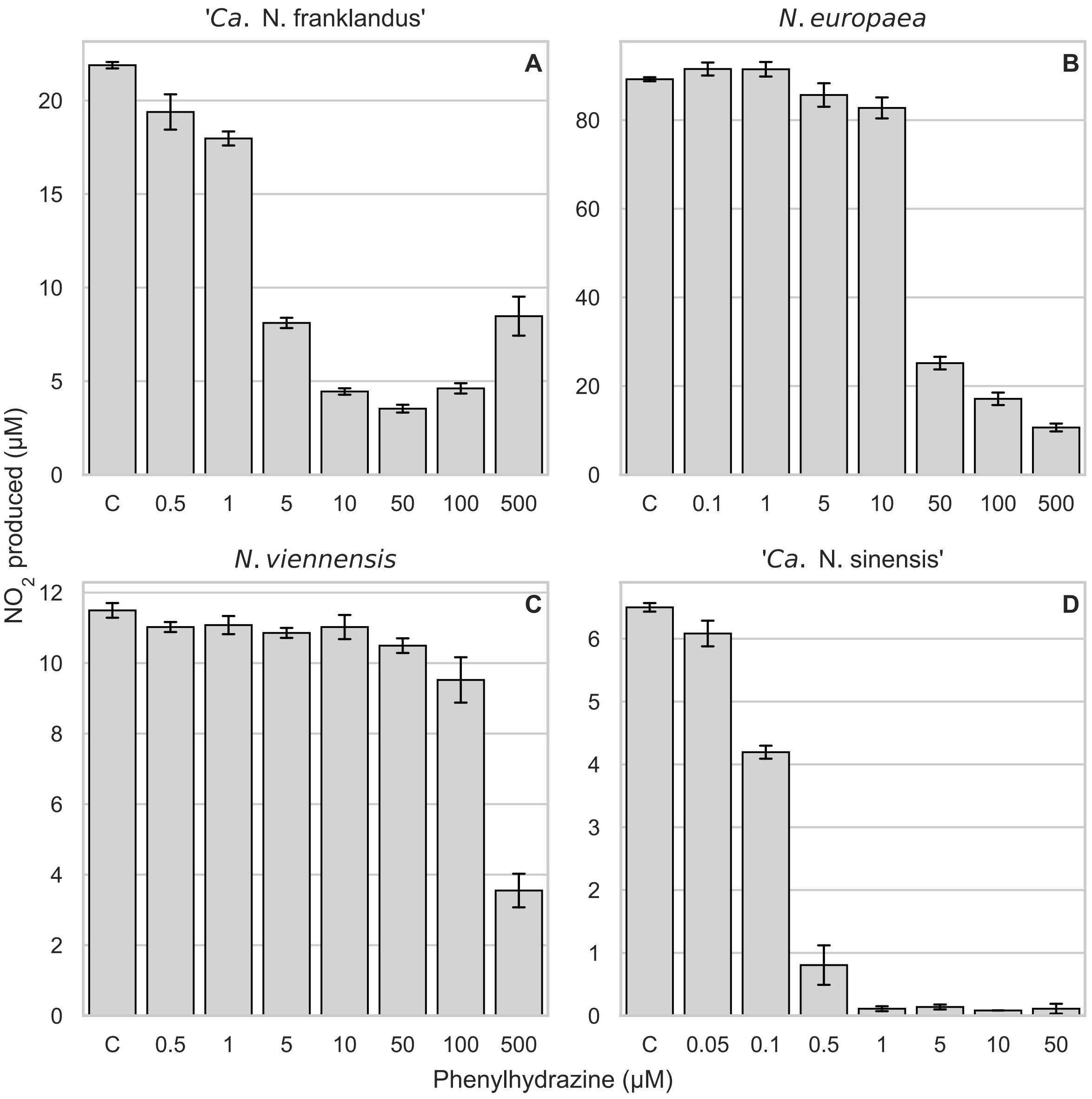

Supplemental Figure 1. NO_2^- production after 1 h incubation with different concentrations of phenylhydrazine using 100 µM NH_2OH as substrate for *N. europaea* and '*Ca*. Nitrosotalea sinensis' and 200 µM for '*Ca*. Nitrosocosmicus franklandus' and *N. viennensis*. NO_2^- was measured 1 h after addition of the substrate. Error bars represent standard deviation (n = 3).

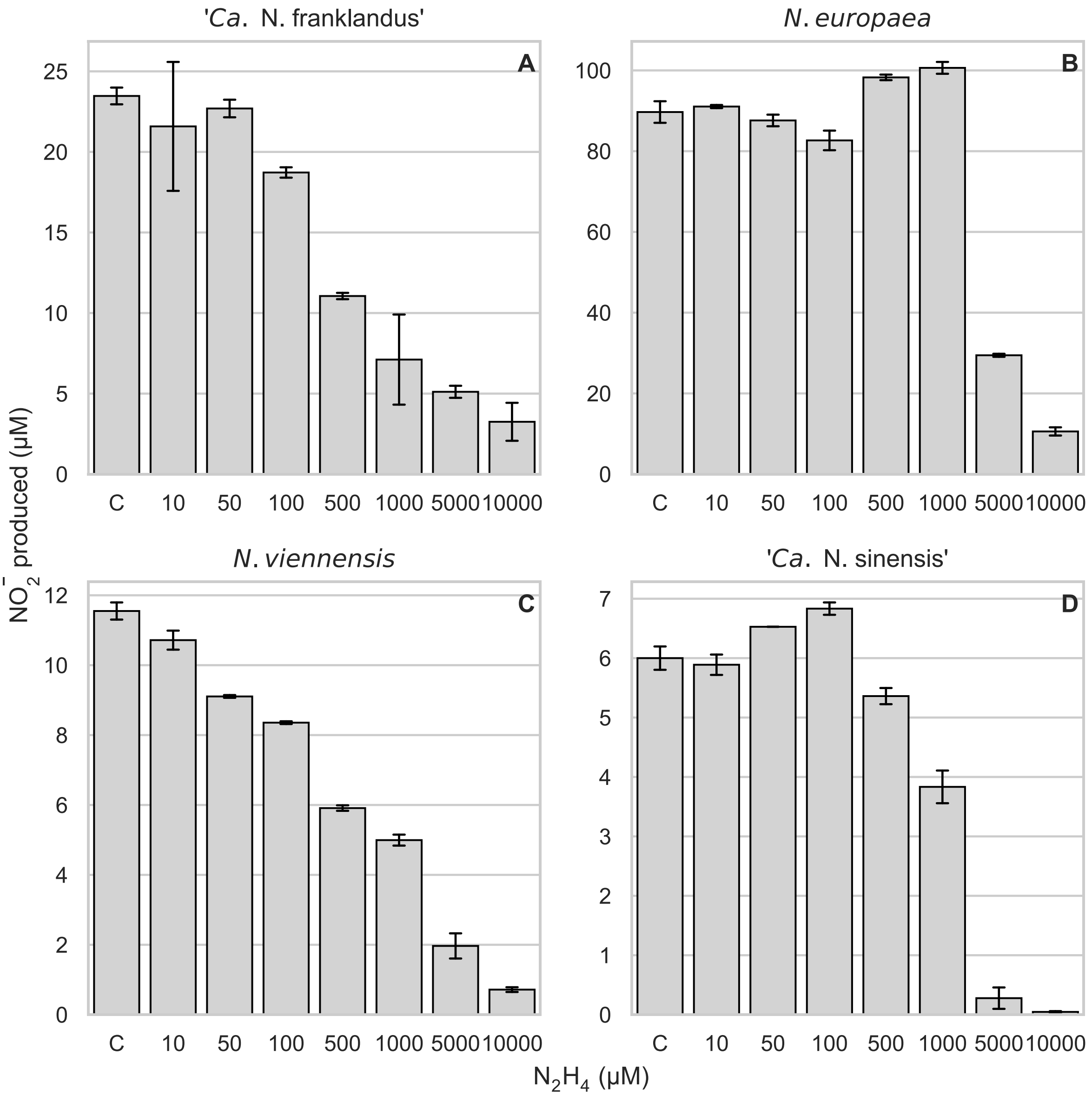

Supplemental Figure 2. NO_2^- production after 1 h incubation with different concentrations of N_2H_4 using 100 µM NH_2OH as substrate for *N. europaea* and '*Ca*. Nitrosotalea sinensis' and 200 µM for '*Ca*. Nitrosocosmicus franklandus' and *N. viennensis*. NO_2^- was measured 1 h after addition of the substrate. Error bars represent standard deviation (n = 3).

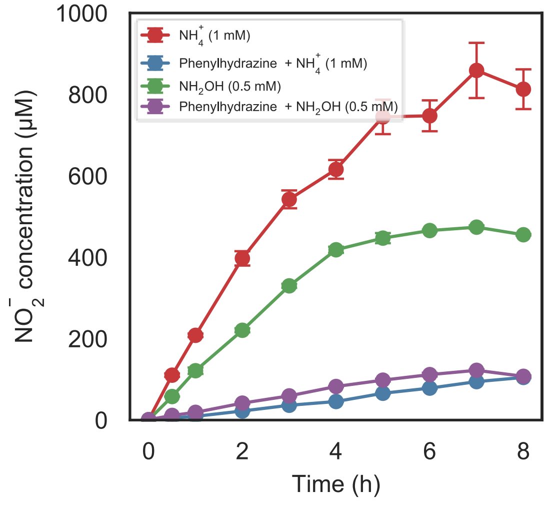

Supplemental Figure 3 – Time course of the recovery of NO_2^- production in *N. europaea* with 1 mM NH_4^+ or 0.5 mM NH_2OH as substrate after removal of 100 µM phenylhydrazine by washing. Error bars represent the standard deviation (n = 3).

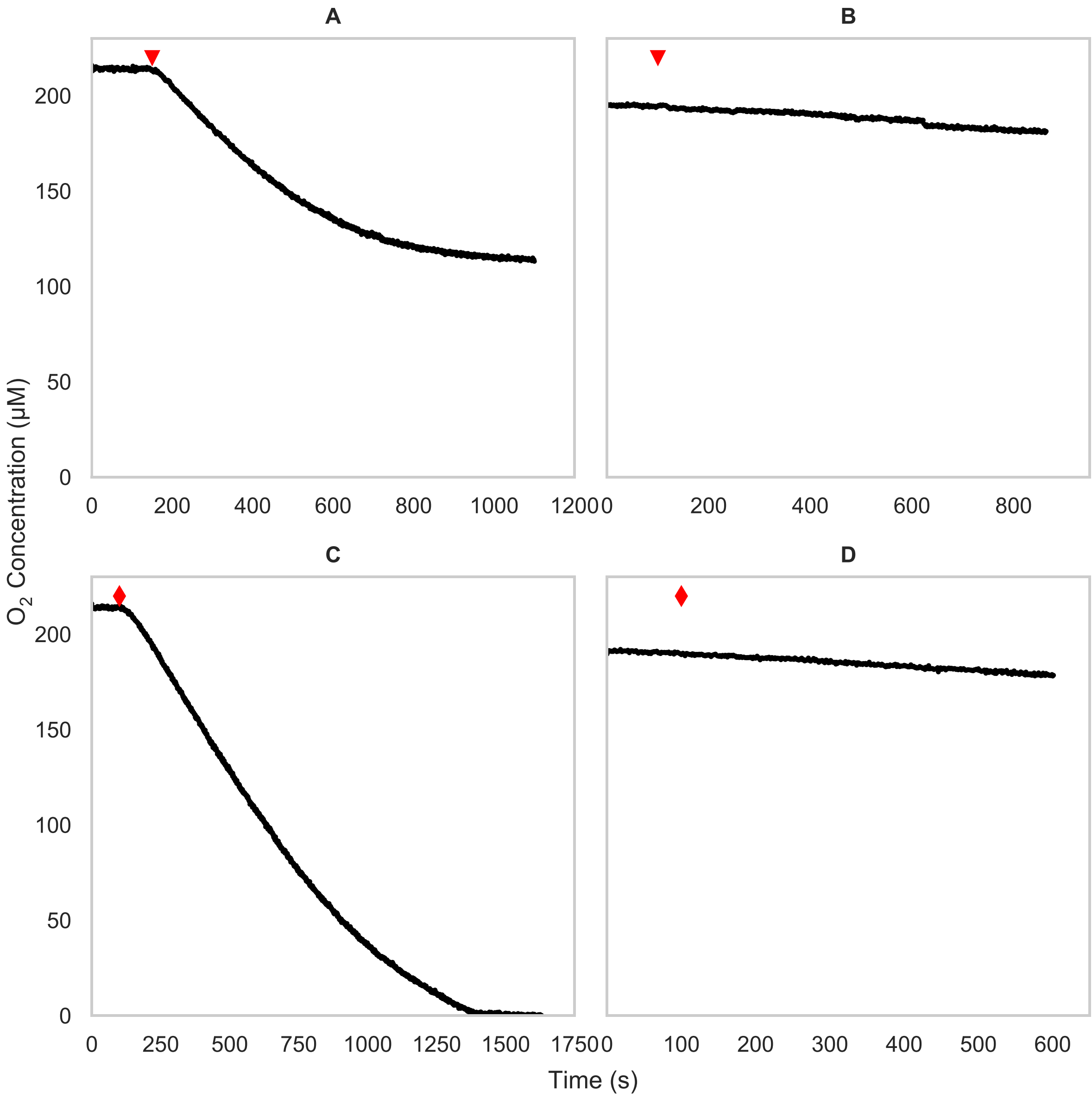

Supplemental Figure 4 – Oxygen uptake measurements by cell suspensions of *N. europaea*. Concentrations of 100 µM NH_2OH (A and B, red triangles) or 600 µM N_2H_4 (C and D, red diamonds) were added. Control cells (A and C) are compared to cells incubated with 100 µM phenylhydrazine (B and D). Experiments were performed at least three times with similar results.

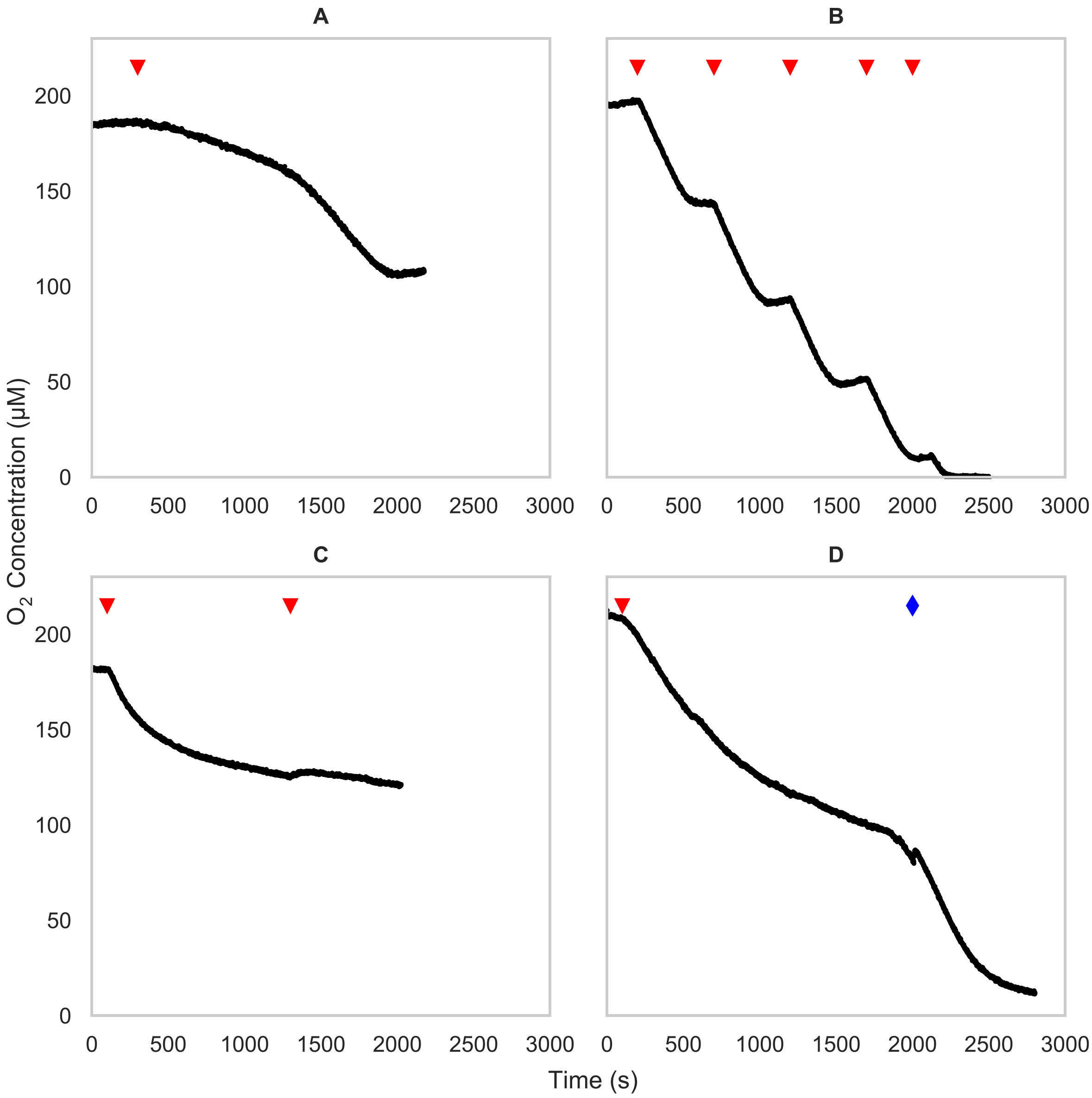

Supplemental Figure 5 – Oxygen uptake measurements by cell suspensions of ‘*Ca*. Nitrosocosmicus franklandus’. A: addition of 400 µM NH_2OH (red triangle); B: repeated spiking with 200 µM NH_2OH (red triangles) until anoxic; C: spiking with 600 µM N_2H_4 (red triangles); D: addition of 600 µM N_2H_4 (red triangle) followed by 500 µM NH_4^+ (blue diamond).

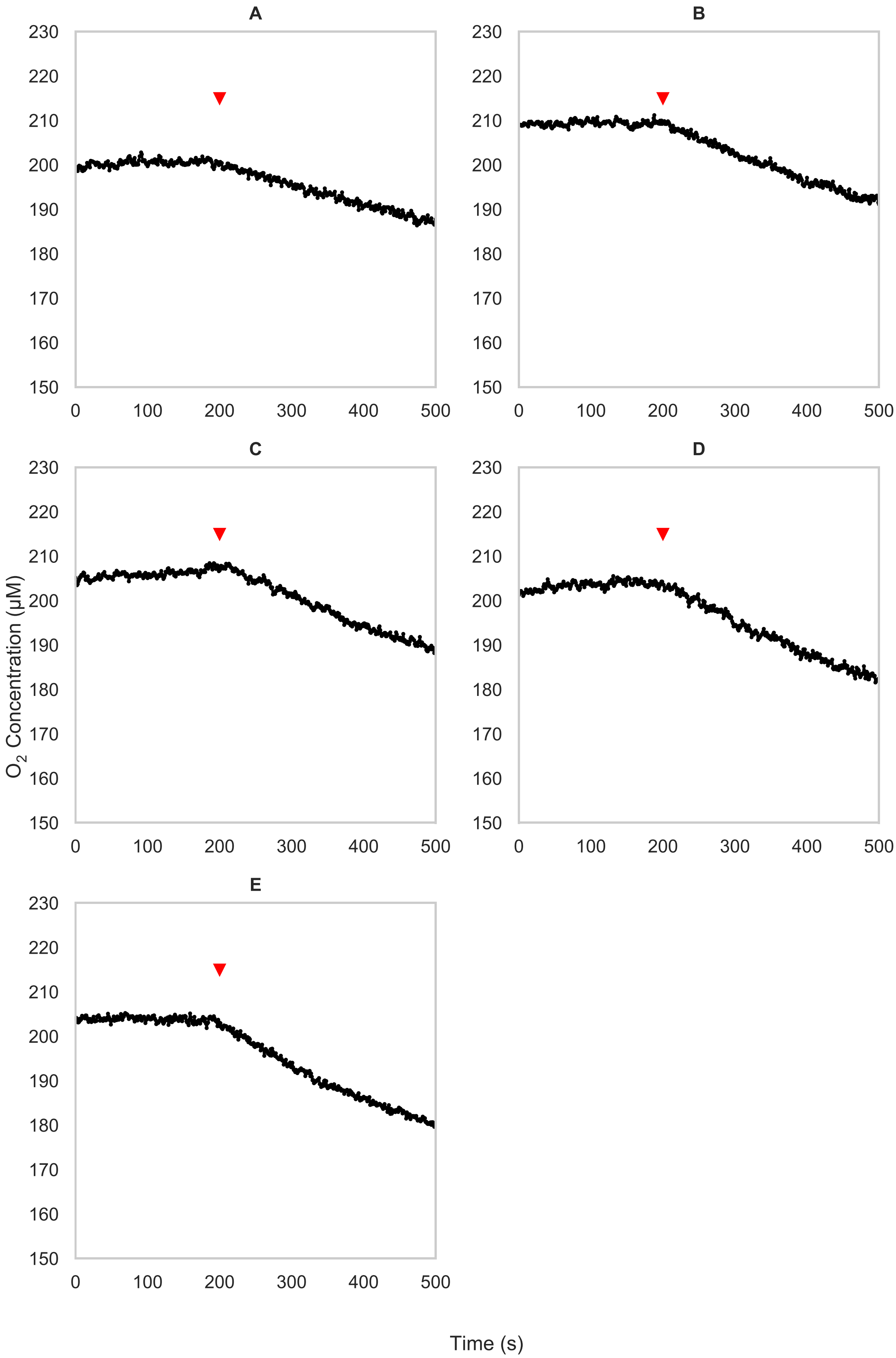

A
B
C
D
E
230
220
210
200
190
180
170
160
150
0
100
200
300
400
500
O2 Concentration (µM)
Time (s)

Supplemental Figure 6 – Initial rates of oxygen consumption in cell suspensions of '*Ca*. Nitrosocosmicus franklandus' after addition of different concentrations of N_2H_4 (red triangles). A: 200 µM N_2H_4; B: 400 µM N_2H_4; C: 600 µM; N_2H_4; D: 800 µM; N_2H_4; E: 1000 µM N_2H_4.

Supplementary Table 1. Experimental design for the end-point inhibition assays

Organism	Ammonia (µM)	Hydroxylamine (µM)	Hydrazine (µM)	Phenylhydrazine (µM)	Results shown
'*Ca.* Nitrosocosmicus franklandus C13'	100	-	-	0.05-500	Figure 1
	-	200	-	0.05-500	Figure S1
	100	-	1-10,000	-	Figure 2
	-	200	1-10,000	-	Figure S2
Nitrosomonas europaea	100	-	-	0.05-500	Figure 1
	-	100	-	0.05-500	Figure S1
	100	-	1-10,000	-	Figure 2
	-	100	1-10,000	-	Figure S2
Nitrososphaera viennensis EN76	100	-	-	0.05-500	Figure 1
	-	200	-	0.05-500	Figure S1
	100	-	1-10,000	-	Figure 2
	-	200	1-10,000	-	Figure S2
'*Ca.* Nitrosotalea sinensis Nd2'	100	-	-	0.05-500	Figure 1
	-	100	-	0.05-500	Figure S1
	100	-	1-10,000	-	Figure 2
	-	100	1-10,000	-	Figure S2

**Supplementary Table 2. Experimental design for the oxygen uptake experiments**					
Organism	Ammonia (µM)	Hydroxylamine (µM)	Hydrazine (µM)	Phenylhydrazine (µM)	Results shown
'*Ca.* Nitrosocosmicus franklandus C13'	-	200	-		Figure 4A
	-	200	-	100	Figure 4B
	-	-	600	-	Figure 4C
	-	-	600	100	Figure 4D
	-	400	-	-	Figure S5A
	-	5 x 200	-	-	Figure S5B
	-	-	2 x 600	-	Figure S5C
	500	-	600	-	Figure S5D
	-	-	200-1,000	-	Figure S6
Nitrosomonas europaea	-	100	-	-	Figure S4A
	-	100	-	100	Figure S4B
	-	-	600	-	Figure S4C
	-	-	600	100	Figure S4D
